# Social Determinants of COVID‐19 Pandemic Control: Participatory Learnings From Everyday Experiences in Cape Town, South Africa

**DOI:** 10.1111/hex.70030

**Published:** 2024-09-16

**Authors:** Frederick Marais, Erna Louisa Prinsloo, Christi Niesing, Petra Bester

**Affiliations:** ^1^ Health through Physical Activity, Lifestyle and Sport (HPALS) Research Centre, Sports Science Institute of South Africa University of Cape Town (UCT) Cape Town South Africa; ^2^ Public Health Somerset Council Taunton UK; ^3^ Citizen Researcher Cape Town South Africa; ^4^ The Africa Unit for Transdisciplinary Health Research (AUTHeR) North‐West University Potchefstroom South Africa

**Keywords:** COVID‐19, human connection, participatory research, public health implications, stigma, subversion

## Abstract

**Introduction:**

As countries adapted their disaster responses to the COVID‐19 pandemic, South Africa responded with an alert‐level risk approach based on epidemiological principles that impacted all societal levels. We explored the everyday experiences of people in Cape Town whose basic needs were met and who could report on the realities of the COVID‐19 pandemic control. Gaining insight into their perspectives contributes to knowledge that can inform policies and practices for managing future public health crises.

**Methods:**

Community‐Based Participatory Research principles guided the design and a series of facilitated dialogues with 18 research participants. The thematic analysis was deepened through two colloquiums with members of an overarching research consortium and a participant reflection workshop.

**Findings:**

The pandemic impacted individuals, their interpersonal relationships, workplaces and communities, leading to societal processes such as stigma, virtue signalling and the subversion of mandates. The public health response had mixed reactions, with useful information about preventive measures being diluted by COVID‐19 denialism and fake news. Health and other authorities were frequently perceived as out of touch with, and unresponsive to, the everyday realities of local communities.

**Conclusions:**

Our study demonstrates the connectedness of people and the need to maintain and re‐establish severed connections. A holistic approach to health care and promotion from social determinants of health and a community‐engaged perspective may significantly increase the outcomes of public health responses.

**Participant and Public Contribution:**

People with everyday experience of the COVID‐19 pandemic—including community members, healthcare workers, case managers, carers and researchers—collaborated on the study design, interview schedule, data interpretation, analysis and refinement of this article.

## 1 Background

After the COVID‐19 pandemic brought the world to a standstill, countries had varied disaster responses. In South Africa, a risk alert‐level mechanism with five adjusted lockdown levels was activated to flatten the curve and control the spread of the coronavirus [1]. Public health control measures in Cape Town, located in the Western Cape province of South Africa, had to contend with discrepancies in health literacy, beliefs, attitudes and behaviours. Individuals and groups, irrespective of socio‐economic status, varied from those who wholeheartedly supported public health measures to those who challenged the existence of the pandemic and the information disseminated by those seen to hold power [2]. This study focused on the everyday experiences of people who faced COVID‐19—either through being in isolation, hospitalised after contracting the virus or in quarantine after close contact with someone diagnosed with COVID‐19—and who lived above the ‘upper‐bound poverty line (UBPL)’ [3]. Gaining insight into their perspectives contributes to knowledge that informs policies and practices to manage future prolonged public health crises. The broad UBPL socio‐economic category includes vulnerable low‐income earners, middle‐income people and the affluent. None of our participants experienced absolute or transient poverty, as they reported being food‐secure, adequately housed, well‐educated and predominantly private healthcare users.

Although the link between poverty and poor health outcomes is well‐reported in scholarly literature, there is limited research on the impact of other socio‐economic conditions on health outcomes during a protracted pandemic such as COVID‐19. In a public health context, we understand that social determinants of health, such as poverty, hunger and overcrowding, create insurmountable barriers to health [4]. This study co‐produces knowledge with people whose basic needs were met, who could speak about their personal experiences with COVID‐19 and who were able to challenge public health measures regarding COVID‐19 control. Participants came from diverse backgrounds, including members of the public, COVID‐19 case managers and contact tracers, transdisciplinary healthcare workers, primary healthcare practitioners, grief counsellors and qualitative researchers.

This article draws on several vital texts to deepen our understanding of the data. These include Link and Phelan's [5] classic work on conceptualising stigma, Walby's [6] work on the theory of crisis and the pandemic and the socio‐ecological model [7, 8] to consider the complexities of interactions among the individual, the group/community and the physical, social and political environments.

## 2 Methods

### 2.1 Study Design

This study formed part of a larger overarching research consortium in the Western Cape that explored a broad range of COVID‐19 experiences among individuals and communities affected by the pandemic to inform public health policy and practice [9]. The focus areas included the study under discussion, an inquiry into university students involved in case management and contact tracing [10], the experiences of frontline healthcare workers and the experiences of healthcare workers involved in end‐of‐life care at a field hospital.

Our study investigated participants' daily experiences during the first three waves of the pandemic (see Table 1 for participant summary). These three waves spanned approximately 18 months, from April 2020 to November 2021 [11]. Community‐Based Participatory Research principles were used to inform a contextually appropriate design for knowledge co‐creation and translation in partnership with the study participants [12]. A series of facilitated dialogues [13] took place between research participants and researchers. More layers of analysis were added at two colloquiums of the overarching research consortium, through regular reflective discussions among the authors of the article (one of whom is a citizen researcher), and through a reflections workshop with participants. In short, this was a collective effort.

**Table 1:** Participant information.

**Focus group**	**Participant number**	**Age**	**Employment sector and status**	**Other identifiers**	**COVID‐19 case or contact**	**Participants' health insurance status**	**Relationship status**	**Comorbidities**	**Hospitalised during COVID‐19 pandemic**
**17 December 2020**	1	40+	Government sector	Religious Graduate	Case	Private	Married with children	Not disclosed	No
	2	40+	Government sector	Graduate	Case	Private	Single	Yes	No
	3	40+	Government sector	Senior management	Contact	Private	Single parent	Yes	No
	4	40+	Government sector	Graduate	Contact	Private	Single	Yes	No
**Individual interviews**									
17 December 2020	5	Not disclosed	Government sector	Graduate	Case	Private	Married, no children	Not disclosed	No
22 December 2020	6	Not disclosed	Government sector	Graduate	Case	Public	Married with children	Not disclosed	No
19 October 2020	7	60+	Private sector	Lives alone	Case	Private	Divorced	Yes	No
22 October 2021	8	70+	Private sector	Graduate	Case	Private	Married	Not disclosed	No
25 October 2021	9	70+	Private sector	Strong social connections	Case	Private	Lives with partner	Yes	Yes
25 October 2021	10	70+	Private sector	Graduate Strong social connections	Case	Private	Lives with partner	Not disclosed	Yes
26 October 2021	11	50+	Private sector	Animal activist	Case	Private	Single	Not disclosed	No
02 November 2021	12	60+	Homemaker	Religious	Case	Public	Married	Yes	Yes
12 November 2021	13	Not disclosed	Private sector	Partner was hospitalised	Case	Private	Partner	Not disclosed	No
17 November 2021	14	50+	Private sector	Religious Graduate	Case	Private	Married	Not disclosed	Yes
22 November 2021	15	Not disclosed	Community activist	Health Activist Religious	Case	Private	Married	Yes	Yes
28 January 2022	16	60+	Private sector	Graduate	Case	Private	Lives with partner	Yes	Yes
07 February 2022	17	80+	Retired	Lives in a retirement village	Case	Private	Married	Yes	No
29 November 2021	18	50+	Government sector	Shares home with tenant	Case	Private	Single	Yes	No

### 2.2 Sample

The initial design was to sample Cape Town citizens living below the UBPL from the COVID‐19 Case and Contact databases in the public health sector. Sampling participants from these case management information sources without their prior consent to be contacted for research participation was deemed ethically inappropriate by the local health authorities. This was due to the heightened sensitivity surrounding a COVID‐19 status at the time of this study. Prolonged difficulties to overcome this hurdle led to the use of snowball sampling, drawing from professional, community and personal networks. The unintended diversion away from people living in poverty to people above the UBPL was serendipitous. It created an opportunity to co‐create knowledge with an under‐researched group by understanding the impact of COVID‐19 on their daily lives and their perceptions of and recommendations for public health measures to manage pandemics.

The authors and their colleagues in the research consortium had diverse backgrounds, including public health, emergency medicine, nursing, clinical care, sociology, anthropology, psychology, social work, transdisciplinary health research and commerce, and were at a similar socio‐economic level to that of the participants.

### 2.3 Data Gathering

Data were gathered through facilitated dialogues [13]. These included one in‐person focus group and 14 telephonic or virtual conferencing discussions with participants. The interviews were conducted in English, Afrikaans or isiXhosa, the main languages spoken in Cape Town, and all transcriptions were translated into English for data analysis.

Drawing from relevant literature, the researchers drafted an initial set of questions to guide the facilitated dialogues. The draft schedule was reviewed by and tested with three community members who volunteered to be interviewed and to give the team feedback. Findings from a workshop with participants of the overarching study and the researchers' collective experience during the early months of the pandemic underpinned the final guideline (Table 1). An iterative process allowed the ongoing evolution of the guideline in response to topics and themes emerging during data collection [12].

We explored the eagerness with which participants talked to others about the realities of the COVID‐19 pandemic, how they navigated quarantine and isolation, and the support they received from others. The final part of the facilitated dialogue focused on personal changes and insights gained from participants' experiences during the COVID‐19 pandemic, as well as the value of public health information, education and support. Participants were asked to reflect on lessons learned and offer recommendations to better prepare for future public health emergencies. The interviews were digitally recorded, translated into English, transcribed and cross‐checked for completeness and accuracy before data analysis.

### 2.4 Data Analysis

The data were subjected to thematic analysis, drawing on the work of Miles and Huberman [14] and Bazeley [15]. All transcripts were reviewed by the research team in February 2023 to verify preliminary findings and deepen our understanding of the participants' experiences. Iterative rounds of knowledge co‐production took place between the article's authors and the overarching qualitative research consortium at two colloquiums. A crucial step was getting feedback from two participants (Participants 15 and 18). They verified the final version of this article by confirming our findings and highlighting the importance of stigma, the negative impact of COVID‐19 denialism and the importance of social connection.

### 2.5 Ethics

The Health Research Ethics Committees of the University of Cape Town and Stellenbosch approved the research proposal. Written or verbal informed consent was obtained from all participants before each interview. Provision was made for participants to receive counselling if they became distressed; however, they did not request this. The confidentiality of participants was ensured by numbering the interviews and anonymising the data. Data were managed by storing it in a secure cloud space that only the research team could access according to the ethical requirements.

## 3 Findings

All 18 participants had first‐hand experience with COVID‐19. Two of the participants had to quarantine as they were in close contact with infected patients. Sixteen participants were infected with the virus, and six of these participants were hospitalised. Twelve of the participants identified as women, and six identified as men. They were asked to share their personal experiences with COVID‐19. The encounters between the research participants and the researchers were steeped in mutual experiences of the pandemic, and this created empathic resonance that benefitted the co‐creation of knowledge.

Almost half of the research participants lived in historically disadvantaged areas, whereas the remainder resided in middle‐income communities. Most participants were older than 40 years of age, financially secure with adequate living conditions, well‐educated (most had tertiary qualifications), employed (a preponderance of participants with management and leadership experience), and had access to private medical services (private medical insurance).

Nearly half of the participants (47%) were healthcare professionals in clinical and/or academic settings. Out of these, seven worked as COVID‐19 case managers, contact tracers or grief counsellors during the peak of the crisis. Their collective transdisciplinary positions—public health practice, social work, dietetics, finance, training, communication and occupational therapy—offered them the advantage of reflecting from public health and personal vantage points. The remaining sample was self‐employed or retired as summarised in Table 1.

During the first three waves of the pandemic, hospitals in Cape Town were under severe pressure, field hospitals were rapidly assembled, and reports of COVID‐19 deaths filled the news and our consciousness [11, 16]. There was no denying that Cape Town was a city in crisis. Walby [6] defines a crisis as an event (in this instance, the pandemic) where the consequences continue for longer than the initial impact, with the crisis having the power to cause significant complex system change. As urged by Walby [6], we paid careful attention to how the crisis cascaded through different spheres of life. We examined the pandemic's influences and impact on individuals, interpersonal relationships, organisational/workplaces, community participation and the public health response. These individuals shared their socio‐economic standing with two‐thirds of adults living in the Western Cape province [16]. This analysis was conducted using the lens of the Socio‐Ecological Model of Bronfenbrenner [7], which demonstrates how social structures of community, society, religion, economics and politics interact and influence interpersonal relationships. Following that, we report on the underlying social processes of stigma, virtue signalling and the embracement and subversion of mandates. The theoretical discussion of stigma is based on the conceptualisation of stigma by Link and Phelan [5]. They identified the following elements in the concept of stigma: labelling, stereotyping, separation, status loss and discrimination, which co‐occur in a power situation that allows the components of stigma to unfold. Tables 2a and 2b provide a summary of the applied theoretical framework, with data analysis guiding the thematic discussion.

**Table 2a:** Summary of data analysis.

Theoretical framework	Theme	Category	Code	Participants
Theory of Crises S. Walby	Social and Emotional	COVID‐19 Crises Vulnerability	The consequences outweigh the event	11
Socio‐Ecological Model Bronfenbrenner	Individual and interpersonal influences	Widespread anxiety		2, 3, 4, 5, 7, 11, 15, 18
				2
		High levels of expressed emotions	Panic, fear, guilt and anger	
		Affected people felt like vectors of disease		11
		Outcasts		2, 11
		Living alone	Boredom, the fear of dying alone, a profound sense of disconnection and humiliation	7
			Psychological impact	18
		History of depression	Confined to home	4
		No physical family support	Feelings of being robbed of contact	7
		Special events	Contact between family members became constrained	2
		Behaviour change in children	Extra cautious and was told not to touch anything	6
		New routines for protection of children	Emotional struggle not to touch before cleansing routine	3
		Tearing apart of close family relations	Heartache of not being able to be with her child and her parents	6
		Self‐actions for social and emotional resilience	Maintaining emotional resilience	1
			Families to rediscover each other	3
		Digital technology	WhatsApp groups facilitated social contact	8, 19, 7
			Telephone conversation support	12
		Spirituality, religion and opportunity for personal growth	Used religion to deal with the crisis	1, 12, 10
			Feeling more resilient, being more comfortable with emotional vulnerability, being good role models, and learning to set boundaries to take better care of themselves	5,9
	Organisational influences	Work environments	Harsh and rejecting	6
		Supporting work environments	Show their emotional vulnerability and receive support	2
			Collegial support – showing true self	3
			Line managers manage anxiety	1
	Community participation influences	Community Action Networks	Growth of dynamic CANS – Community Kitchen	15
			Impediment of existing structures – Absence of PPE­ health risk	15

**Table 2b:** Summary of data analysis.

Theoretical framework	Theme	Category	Code	Participants
	Public Health Response Influences	Sufficient public health information	The provincial health service was praised – Information about the pandemic	1, 2, 3, 4, 9, 10, 11, 12, 13, 17
			Case and contact tracing	
			Counselling services	
			Grief counselling	
			Guidance by volunteer physiotherapist	
		National Coronavirus Command Council	Heavy‐handed mandates during hard lockdown – millions of South Africans living in overcrowded informal dwellings	7, 11
			The pandemic highlighted what a problem alcohol abuse and domestic violence are in South Africa – Reduced road accidents and incidents of domestic violence	11
Defining Stigma Link and Phelan	Stigma and virtue Signalling	Secrecy to reduce stigma	Fear	15
			Distrust and fear of being shamed	3, 15, 6
			Omission to disclose in community	1, 11
			Omission to disclose in workplace	5
			Conflict between workplace behaviour and behaviour in community	3, 7
		Personal reputation	Reputational damage and being perceived as irresponsible.	5
			Guilt about causing harm to others	2
			Anxiety and guilt about being a bad citizen and potentially harming others	18
	Embracement and Subversion of Mandates		COVID denialism and anti‐public health sentiments	13
			Noncompliance to wearing of masks and social distancing	5
			Noncompliance of social gatherings	1, 4

### 3.1 Individual and Interpersonal Influences

Essential workers returning from work feared infecting loved ones. Children were not allowed to hug their parents until the parents had gone through an elaborate cleaning ritual of changing out of work clothing and having long showers (Participant 3). Groceries and deliveries were wiped down with sanitiser or placed in the sun for hours. Home surfaces were regularly wiped down. The bedding and sleeping quarters of deceased relatives were subjected to deep cleaning, with some families fretting about their inability to afford professional cleaners. Anxiety was keenly felt.

And then when COVID hit our neighbourhood … in the back streets, it became havoc. It was like a match—and then the flame—and then it started spreading like wildfire. And people started gossiping. Did you hear? The ambulance came, and they took three families. And that was scary.

(Participant 15)

Living alone is unusual in resource‐poor communities where overcrowding and multigenerational households are commonplace, but not so in better‐resourced homes. A participant who lived on her own gave a tearful account of her fear of isolating alone.

I've got co‐morbidities. I live alone. I don't have parents. What the hell's going to happen to me? So, it was one of the most stressful periods for me, because, who's got my house key, who's going to be able to drop things off for me? Fourteen days is a hell of a long time, especially if you're alone, and so that was the scariest for me.

(Participant 4)

Our research participants explained the physical, social and emotional distance the COVID‐19 pandemic created in families. Plans for important family events such as weddings and landmark birthdays had to be postponed, drastically altered or cancelled. Vulnerable relatives were avoided for fear of infecting them. Children were shielded emotionally and physically from COVID‐19 by their parents, which impacted their confidence and changed how they engaged with their communities.

Sustaining social contact was essential for maintaining emotional resilience. Examples abound of family, friends and colleagues who kept in touch with the ill person, ran errands, assisted with shopping and dropped off masks, sanitiser, medicines and nutritional supplements. Families rallied to cope with changed circumstances and quickly adjusted living arrangements.

Everybody phoned. People knew we didn't want to be on the phone, but they needed to be reassured. Even now, people are still contacting us to make sure we're okay. I think your friends carry you through this thing. It's important to tell the people who care about you. Don't underestimate yourself and assume that people don't care.

(Participant 12)

Distance made little difference; family and friends could stay connected with sick relatives. A participant cited the example of a friend in a distant province who maintained contact with her via WhatsApp.

My one friend phoned me daily, she lives out in Mpumalanga. And she said to me, you don't need to talk because there was a time when I couldn't talk. I had all these sores in my mouth and throat, and it was very painful, and I was out of breath. So, she said, I must just listen so that she just knows I am still okay. She phoned me three times a day just to hear if I'm still alive.

(Participant 19)

Digital devices provided human connection at a time when people felt intensely lonely and offered endless entertainment during periods of isolation.

In trying to make sense of their COVID‐19 pandemic experiences, some participants turned to religion or spirituality or viewed the pandemic as an opportunity for personal growth.

I feel a bit stronger and that I can cope better in the work environment and at home. And wherever I go, perhaps, to know where my boundaries are and what I need to do. And then also to realize that if something does happen, that is not totally in your control, and you have to trust others to help you. I feel stronger in that sense, you know, I think I feel like I've grown.

(Participant 5)

When faced with uncertainty, religious participants almost invariably invoked God's help. One participant described how isolation resulted in a mystical experience that helped her cope with loneliness (Participant 12). A Muslim mother used the COVID‐19 crisis as a teachable moment for her children by praying in English rather than Arabic, so the children could understand what she was saying (Participant 1). Religion also served to strengthen attachments, convey care and help some to think differently about what is essential in life.

### 3.2 Organisational Influences

Work environments were harsh and rejecting of those who tested positive. Emotions ran so high that some workers had to be sent home to regain their equilibrium or became verbally abusive towards colleagues, overstepping interpersonal boundaries in a manner unheard of before the pandemic (Participant 3). Healthcare workers were punished overtly and covertly, especially those diagnosed early in the pandemic. The assumption was that they somehow acted irresponsibly despite their medical knowledge.

…in terms of my work, I think that was my worst experience. I felt I wasn't treated fairly because I was positive … I was judged because I got it from my dad and my dad got it from my uncle. The first question was, why was your dad by your uncle?

Why were you by your parents? These are my parents, I'm the only daughter, they are elderly, and I have to look after them. So, if I can't go to the house, who is going to help them?

(Participant 6)

The participant believed that she was being shunned because she was seen as irresponsible. Consequently, she was ignored for 5 days before anyone asked about her well‐being.

Although we found many examples of stigma and rejecting behaviour, this was counterbalanced by families, friends and communities who displayed extraordinary kindness (Member Reflection Workshop). There is also a strong sense that teams in the workplace matured as the pandemic progressed. Camaraderie grew along with the pandemic.

I can vouch for this because I'm here at 7 o'clock. So, before we get coffee or whatever, ‘how are you?’ Even just for five or 10 minutes, catch up, even if you are talking nonsense or laughing. Whoever comes in the door at seven o'clock we will clap to make that person feel appreciated at work. One of my colleagues cried one morning and she said, ‘you make me feel that I want to be here’. For us, we didn't realise we had an impact in that way. So, for us that morning, just to chat and to talk nonsense, to lift our spirits, even if we just listen to music or just talk, make a joke.

(Participant 2)

Over time, the venting of negative emotions moderated in the workplace, and more support was offered to affected colleagues.

### 3.3 Community Participation Influences

This study helped us understand how pockets of citizen activism and resistance spontaneously emerged. A critically enabling role was offered by volunteers, community groups and networks. The provincial health response was bolstered by volunteers who did case management, contact tracing and counselling [11, 12]. Cape Town experienced the rapid growth of dynamic Community Action Networks [17] in response to the vacuum left by cumbersome formal structures that were impeded by bureaucracy and unable to mount a rapid response. Altruistic individuals, groups and networks mobilised human and material resources to set up community kitchens in resource‐poor areas. These community kitchens offered more than food; they also provided social support and health education, strengthening broader determinants of health. Unfortunately, their efforts were not always encouraged by overzealous law enforcers. Some independent health activists reported difficulty convincing law enforcement that they should be permitted to run community kitchens. Driven by her inner moral code, one participant sidestepped law enforcement. She provided a service without personal protective equipment or a clear understanding of preventive measures. Consequently, she and her husband became seriously ill with COVID‐19 (Participant 15).

### 3.4 Public Health Response Influences

Most of our participants felt that there was sufficient information on how to limit the spread of the virus and that the public health system had met its remit towards citizens. COVID‐19 denialism and fake news damaged public health efforts (Member Reflection Workshop), and criticism was levelled at the National Coronavirus Command Council (NCCC) and some heavy‐handed mandates during hard lockdowns. Hard lockdowns were out of step with the millions of South Africans living in overcrowded conditions, the need to preserve livelihoods and those living independently without adequate social networks [18].

It has made me look at governments and the way they handle things and the way they do things. It's made me realize they are very removed from their people. You shut people's lives down and it's easy for governments to do knee‐jerk reactions without really understanding the impact on people. It has left me and many people with less trust.

(Participant 11)

The pandemic illuminated the severity of alcohol abuse and domestic violence in South Africa. The decision to impose strict curfews and ban the sale of alcohol during the hard lockdowns divided public opinion: some argued that these measures curtailed personal freedoms, whereas others approved them wholeheartedly [1].

When we stopped alcohol, and we saw the absolute response. We saved lives during COVID. We did not lose them. We saved them in terms of road accidents and domestic violence because once alcohol was not available it was amazing to see the difference.

(Participant 11)

Hospital emergency rooms benefitted from a drastic reduction in violence‐related casualties and road accident victims, but numbers quickly returned to previous levels once the alcohol ban was lifted [19].

### 3.5 Stigma and Virtue Signalling

As the virus spread, communities started panicking, and the stigma grew along with the fear (Member Reflection Workshop). Stigma reduced as the pandemic progressed; as more people were diagnosed, COVID‐19 became normalised, and the public gained a better understanding of the virus. Attitudes were harsh during the early phases of the pandemic.

The hardest thing with COVID was how other people perceive you. People get hostile and so you must be careful living in a block of flats. I chose not to tell anyone because my feeling was if you did, suddenly you become this leper the minute you coughed. I felt people with COVID were almost looked at as lepers. They were treated badly by neighbours and by other people.

(Participant 11)

Link and Phelan [5, pp. 366–375] in conceptualising stigma, describe the hallmarks as labelling (‘othering’), stereotyping (e.g., dismissing some people as irresponsible), marginalisation (separating undesirable people from larger society), status loss and discrimination. The practice of isolating symptomatic people and their close contacts laid the groundwork for stigmatisation. Although this measure was crucial to protecting citizens, separating ‘ill people’ from ‘healthy people’ meant that those removed from social contact were frequently shamed and treated with hostility.

Power always plays a role in stigmatisation [6], irrespective of whether the antagonist is the family, a community group, an employer or the management of an apartment complex. Secrecy is a typical response to stigma that fuels more shame, limits help‐seeking behaviour and affects mental health. Thomas Scheff [20, pp. 1–14] writes about the emotional–relational world and posits that humans go out of their way to hide their shame. Shame is relational, associated with the threat of social disconnection, and is a significant means of social control [20, pp. 9–10].

Pinning down the source of infection became a preoccupation for many. Underlying much of this was concern about reputational damage and being seen as irresponsible. Participants reported overwhelming feelings of guilt about potentially causing harm to others and the burden of being responsible for the death of others. ‘…if they die, I'm going to be the cause. I'm going to have to live with myself’ (Participant 2). Virtue signalling was commonplace, especially important among those infected lest they be considered irresponsible. There was more embarrassment about causing harm to people outside the family. Individuals used virtue signalling as a coping mechanism to manage their embarrassment by insisting that despite taking great care, they nevertheless became infected.

Yoh! I was shocked, I was upset because I was very careful not to get it. I protected myself by wearing my mask, sanitising, and not associating with others. And whenever I spoke to somebody, I always asked them, ‘Do you have any cases at the office? Do you keep social distance? Do you wear your mask?’. I always took all the precautions not to get it. I was shocked, I was disappointed, I was scared. I was very scared.

(Participant 18)

Neil Levy [21], a philosopher, argues that rather than viewing virtue signalling as ‘moral grandstanding’, we should recognise that it enables cooperation and a willingness to act for the greater good. Levy sees virtue signalling as virtuous unless underpinned by dishonesty and excessive moral outrage.

### 3.6 Embracement and Subversion of Mandates

In the early days of the pandemic, people were petrified of getting infected or dying, more inclined to catastrophise the potential dangers of COVID‐19 and more ready to embrace mandates. Initially, there was a heavy emphasis on surface cleaning and hand washing, and the NCCC was slow to adjust regulations as scientific understanding grew (Colloquium 2) [1]. Towards the end of 2020, COVID‐19 became more normalised, and people were less anxious about getting sick. Some participants stood firmly behind the public health effort and remained resolute in their support for control measures. Others, irritated by what they saw as unreasonable restrictions and/or motivated by COVID‐19 denialism and anti‐public health sentiments, were more inclined to subvert mandates. The sad irony was that towards the end of 2020, South Africa was poised for the unfolding of its deadliest wave at the point when people were becoming more cavalier about preventive measures [1].

Being deprived of human contact created emotional vulnerability, and some participants could not resist the need for social contact when regulations prohibited it. Transgressive behaviour occurred at work, in public spaces and in private. An overwhelming number of research participants cited examples of noncompliance with COVID‐19 pandemic mandates.

Compliance with COVID‐19 pandemic control measures at work does not necessarily mean compliance at home. There can be tension between professional and personal selfhood. A participant raged about trying to educate the public. However, once in her private domain and away from her professional role, the heartbreak of being unable to visit a dying relative pushed her to lie about being a COVID‐19 contact so she could get past her gatekeeping spouse. She understood that he wanted to protect his wife but that these were strange times—a time of COVID‐19, a time of saying goodbye to a fading aunt and an emotional time (Participant 3).

## 4 Recommendations

The lockdown of the general population, quarantine of close contacts and isolation of people with COVID‐19 ruptured social connections.

**Figure 1:**
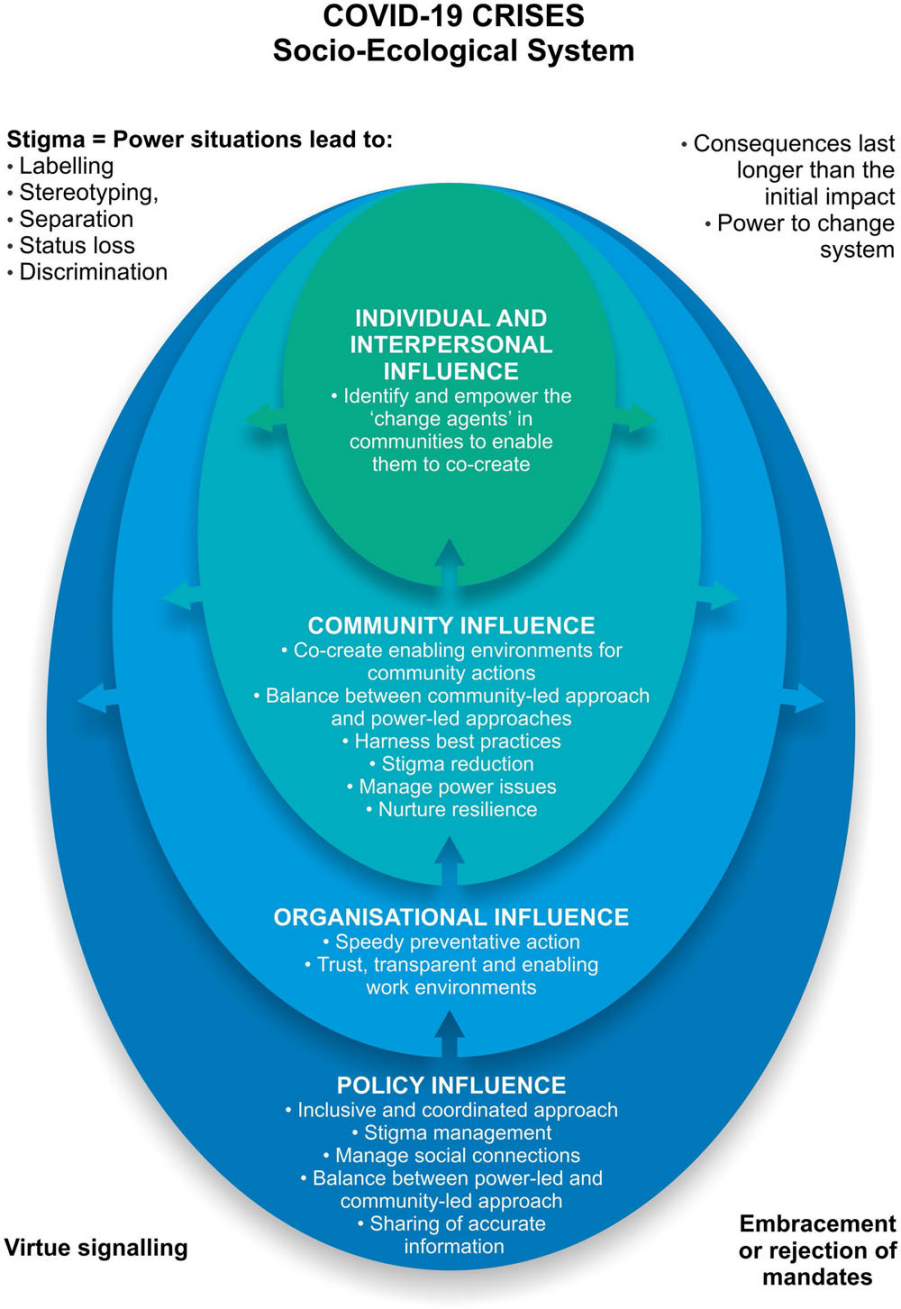
Application of the socio‐ecological model in the system.

A holistic approach to health care and promotion interventions from a Social Determinants of Health perspective [22] and with community‐engaged principles [13] may significantly increase the outcomes of the interventions. The socio‐ecological model explores the interplay between the various social determinants of health from the individual to the family, the community and the larger society and explores the complexities of these components of a system. The model is applied to the study results to develop a contextual model for further exploration of such interventions in a South African setting [23]. Figure 1 provides an applied infographic of the socio‐ecological model to the data analysis.

### 4.1 Individual and Interpersonal Influences

In preparing a community for a crisis, all levels of society can be influenced when the *individual is empowered*. Therefore, the recommendation is to start with the *healthcare worker, the change maker and the individual who plays a leadership role* in a community. This includes the individuals enabling the Community Action Networks [24]. Healthcare workers need more insight into the crisis theory [7, 25] and how to rapidly adjust to working under crises. Credence needs to be given to the agency of frontline health workers. They need to be given leeway to introduce their novel responses to the crisis, as managers were seen to be overburdened and largely unresponsive, especially during the early phases of the pandemic.

### 4.2 Community Engagement Influences

An effective community‐engaged response can only be complete with the wholehearted inclusion of volunteers and informal initiatives. Public health practitioners need more skills in applying community participatory principles [13]. We improve our chances of recovering from a crisis and bringing about change to our health system by accepting the contribution made by citizen‐based initiatives. Adopting a bottom‐up approach in co‐creating community‐engaged public health measures for the speedy withdrawal from public spaces (including workspaces) can successfully be implemented in environments where stigma is adequately managed. Intentional management of power dynamics in communities might increase adherence.

### 4.3 Organisational Influences

Workspaces must quickly implement mandates (e.g., social distancing and masks), decongest offices, pay close attention to adequate ventilation, replace in‐person meetings with virtual conferencing and ensure a good supply of personal protective equipment for all staff, including low‐status staff. Top‐heavy and rigid management structures present a high risk during a crisis. Work environments embedded in trust promote transparency.

### 4.4 Public Health Influences

Coordinating all national, provincial and local agencies is crucial during a crisis. It was felt that the Department of Health did not always receive adequate support from police and social development services.

From a public health perspective, a sophisticated approach to stigma management during a major crisis is required. In interrogating stigma, we must always be aware of power dynamics because power drives stigmatisation. South Africans have a long history of not taking kindly to imposed power; therefore, care must be taken in how top‐down bodies such as the NCCC are framed and how mandates are imposed.

Practice evidence‐based medicine in mandates must be modified speedily as scientific understanding grows. Nonsensical regulations should have been put aside much sooner to prevent cynicism and noncompliance. Although good hygiene undoubtedly limited the spread of other infections, it also led to high levels of anxiety.

Accurate information must be disseminated timely and through various channels to ensure the correct information is readily available.

## 5 Concluding Remarks

This participatory study presented us with a unique opportunity to co‐produce knowledge about how our healthcare system responded during a profound health crisis, how people made sense of the pandemic and how research findings can be used to advance our healthcare system.

In the early days of the pandemic, people with COVID‐19 became devalued because they presented a risk to health services and were seen as transmitters of disease and possibly death. That early stigma lessened as more people were diagnosed, the virus became better understood and health services became more competent and empathic in meeting needs.

The rupture of social relationships stood out as a major stressor, with ample evidence of emotional tumult in response to perceived danger, profound feelings of loneliness and ongoing efforts to gauge personal risk, especially in those participants with comorbidities. Many felt a deep sense of isolation—from the young to the old, and from extended families to single people living on their own. This isolation resulted in a loss of emotional equilibrium—a sense of losing balance—especially for those isolated on their own and those kept away from sick or dying loved ones. Some of the emotional distress was lessened by dogged efforts to remain socially connected, the reliance on digital devices, drawing on spiritual practices and the subversion of mandates.

Volunteers and community groups made a noteworthy contribution to the collective effort. Community participation and the accounts of affected people are powerful in challenging healthcare practice and policy. In the future, our policies and practices must be grounded in evidence and community engagement to ensure compassion and kindness while meeting public health goals such as efficiently containing the spread of infection and caring for those affected. This care cannot be nonresponsive to the emotional needs of people and must incorporate mental health as a fundamental part of service delivery.

Overprotectiveness found expression in harsh national mandates and the heavy‐handed treatment of essential workers. Over time, paternalism created resistance and resulted in resentment and the subversion of mandates. It is not easy to retract mandates once they have been issued. Therefore, mandates must be based on rigorous scientific knowledge and make sense, and their impact on interpersonal relationships must be carefully considered—especially in a context where there is an active distrust of authority. It is not helpful to sever social connections and damage relationships, resources and livelihoods. We must find innovative ways of limiting infection without being overly controlling.

## Author Contributions

**Christi Niesing:** conceptualisation, investigation, funding acquisition, writing–original draft, methodology, visualisation, writing–review and editing, formal analysis, project administration, resources. **Frederick Marais:** conceptualisation, investigation, funding acquisition, writing–original draft, methodology, validation, writing–review and editing, data curation, resources. **Erna Louisa Prinsloo:** investigation, writing–original draft, methodology, validation, writing–review and editing, formal analysis. **Petra Bester:** conceptualisation, investigation, funding acquisition, writing–original draft, visualisation, writing–review and editing, resources.

## Ethics Statement

Ethical approval was obtained from the HREC at the University of Cape Town and Stellenbosch University.

## Consent

Participants provided written consent to partake in the study.

## Conflicts of Interest

The authors declare no conflicts of interest.

## Data Availability

Data are available on request due to privacy/ethical restrictions. The data that support the findings of this study are available on request from the corresponding author. The data are not publicly available due to privacy or ethical restrictions.
